# Non-invasive central aortic pressure measurement: what limits its application in clinical practice?

**DOI:** 10.3389/fcvm.2023.1159433

**Published:** 2023-05-26

**Authors:** Alejandro Diaz, Yanina Zócalo, Federico Salazar, Daniel Bia

**Affiliations:** ^1^ Instituto de Investigación en Ciencias de la Salud, UNICEN-CCT CONICET, Tandil, Argentina; ^2^ Departamento de Fisiología, Facultad de Medicina, Centro Universitario de Investigación, Innovación y Diagnóstico Arterial (CUiiDARTE), Universidad de la República, Montevideo, Uruguay; ^3^ High Blood Pressure Section, Cardiology Department, Hospital Privado de Comunidad, Mar del Plata, Argentina

**Keywords:** aortic blood pressure, calibration, non-invasive assessment, oscillometric method, blood pressure measurement, central blood pressure measurement

## Abstract

The following article highlights the need for methodological transparency and consensus for an accurate and non-invasive assessment of central aortic blood pressure (aoBP), which would contribute to increasing its validity and value in both clinical and physiological research settings. The recording method and site, the mathematical model used to quantify aoBP, and mainly the method applied to calibrate pulse waveforms are essential when estimating aoBP and should be considered when analyzing and/or comparing data from different works, populations and/or obtained with different approaches. Up to now, many questions remain concerning the incremental predictive ability of aoBP over peripheral blood pressure and the possible role of aoBP-guided therapy in everyday practice. In this article, we focus on “putting it on the table” and discussing the main aspects analyzed in the literature as potential determinants of the lack of consensus on the non-invasive measurement of aoBP.

## 1. Introduction

When a subject lies down, the diastolic and mean blood pressure (DBP, MBP) remain relatively constant in all body arteries. In contrast, in general terms, systolic and pulse pressure (SBP, PP) are higher in peripheral than central arteries. In fact, brachial SBP and PP (bSBP, bPP) was greater than aortic SBP and PP (aoSBP, aoPP), respectively, for the same MBP and DBP (1). This phenomenon, called “systolic and pulse pressure amplification” (SBPA, PPA), is related to arterial characteristics, such as lengths or distances between measurement sites, levels of arterial stiffness, and wave reflections along the arterial tree, etc. (1, 2). Consequently, the relationship between SBP or PP levels measured at the brachial artery (BA) and measured at the aortic level is highly “subject-specific,” requiring individualized assessments. It is impossible to know a subject's aoSBP or aoPP levels simply by knowing the bSBP or bPP levels.

While differences between blood pressure (BP) levels recorded in different arteries have been known for several hundred years, it is only relatively recently that attention has been paid to them for clinical purposes. Although not without limitations (e.g., under- and over-estimation of bSBP and bDBP, respectively) (3), the possibility of measuring brachial BP (bBP) in a non-invasive, innocuous, low-cost, and relatively operator-independent approach has been one of the great “milestones” in medicine. Nowadays, although it is known that there are differences in the levels of bBP determined with different measurement techniques (e.g., oscillometric vs. auscultatory), bSBP and bDBP are considered ´independent´ of the selected method of registration. In contrast, without ignoring the value of bBP, it is only in the last 20–30 years that research has begun to evaluate the potential usefulness of adding aortic BP (aoBP) non-invasive determinations for medical purposes. Due to its proximity to the heart and the brain, aoBP could provide crucial information on the real levels of ´dynamic load´ faced by the ventricular walls or the mechanical stress suffered by the cardiac and cerebral vessels, etc. (4). However, unlike the already more consensual techniques for recording bBP, several methodological issues remain to be addressed before aoBP measurement is fully integrated into clinical decision-making and of practical benefit to patients. In this article, we discuss the main aspects that deserve to be analyzed to move towards a near future in which aoBP measurements are included in clinical practice.

## 2. Methodological issues

At least four methodological aspects remain under discussion, and without reaching an unquestionable consensus:

1. the best technology or mathematical approach to quantifying aoBP,
2. the best arterial recording site,
3. the best way to calibrate the signals,
4. the existence of (poorly studied) proportional errors.

Additionally, it should be considered that many of the technologies or approaches proposed to measure aoBP have not been validated directly against invasive methodologies and/or have not been validated for use in specific populations (e.g., children, pregnant women) and conditions (e.g., validated for ambulatory studies and/or performed during cardiopulmonary exercise test, in which human body position, movement, adaptative responses and/or homeostatic adjustments would modify the aoBP/bBP relationship). Furthermore, methods have recently been proposed to “estimate” aoBP based on simplified approaches [e.g., estimating aoSBP from knowing bMBP and bDBP (e.g., aoSBP = bMBP^2^/bDBP) or applying equations from population-based studies that relate aoSBP levels to individual characteristics]. However, estimating is not measuring, and consequently, in this short article, we will not focus on these additional points.

### 2.1. The best technology or mathematical approach to quantifying aoBP

Concerning the first point, currently, the non-invasive estimation of aoBP is done using a variety of commercial devices that differ: (i) in the principles considered for recording the pulse waveform or surrogate signals (applied technology), (ii) in the model or mathematical analysis applied, and (iii) in the arterial recording site (5–8). Most devices use oscillometry/plethysmography (e.g., cuffs placed at BA level), applanation tonometry [e.g., radial artery (RA) recordings], or vascular ultrasound [e.g., common carotid artery (CCA) recordings] to obtain RA, BA or CCA arterieś pulse waveforms. Then, from the acquired waveforms, and after their calibration, the devices quantify aoBP ´directlý (e.g., direct calibration of CCA waveforms) or ´indirectly,´ for instance, applying generalized transfer functions (GTF), low-pass filters (e.g., N-point moving average, NPMA) or wave analysis algorithms [e.g., detection of the second shoulder in the RA waveform (P2)] (2, 5). Differences between devices and methodological approaches could determine discrepancies in the non-invasively obtained aoBP (6, 7). However, this is still a controversial issue. Results from our group indicate that using different technologies or applying different methodologies would not be among the main determinants of differences in aoSBP levels, at least in comparative terms, concerning the errors related to the recording site or the calibration method. In fact, after recording in the same arterial site and calibrating in the same way (e.g., with the same bBP values), no differences were found (i) when applying different technologies on the same artery (e.g., CCA tonometry vs. ultrasound) or (ii) different mathematical approach applied on the same pulse waveform (e.g., RA tonometry). In this regard, when calibrating using an identical approach [e.g., a form factor (FF) = 33%] and bBP values, aoSBP levels were 120 ± 4 mmHg when recording with CCA tonometry and applying GTF, 122 ± 4 mmHg when recording with CCA tonometry without applying GTF, and 120 ± 3 mmHg when applying CCA ultrasound (invasive levels of aoSBP were 131 ± 4 mmHg). On the other hand, using RA tonometry and identical calibration method and values, aoSBP levels were 121 ± 3 mmHg when applying radial-to-aortic GTF, 122 ± 4 mmHg when determining P2, and 120 ± 3 mmHg and 122 ± 3 mmHg when applying low-pass filters (NPMA 4.0 and 4.4, respectively) (8). These differences (∼1–2 mmHg) are irrelevant clinically and statistically.

### 2.2. The best arterial recording site

Regarding the second point, the arterial recording site could be one of the main determinants of the ability of a non-invasive method to assess aoSBP values. According to recently published data, there is a hierarchical order in terms of the ability to quantify real aoSBP values: CCA>BA>RA (8). Consequently, the closest approximation between invasively and non-invasively measured aoSBP was obtained when considering CCA recording (regardless of the method used) (8). This could be related to the fact that CCA records do not require the use/application of specific wave propagation models (e.g., GTFs), generally derived from population studies and which could not adjust to the specificity of the patient evaluated but assume similarity in BP levels and waveforms between the aorta and CCA (due to their anatomical proximity). Consequently, direct records from CCA should be attempted (prioritized) when quantifying aoSBP values non-invasively (8). However, high-quality CCA records are not always possible to obtain (e.g., in very thick necks, in subjects with respiratory disorders, in neonates or infants), and alternative recordings are necessary. In these cases, peripheral waveform records can be used to obtain (estimate) aoSBP. Our results show that the degree of agreement between data from peripheral waveforms analysis and the obtained invasively would be lower than when evaluating CCAs arteries (8).

Additionally, it should be noted that when obtaining aoBP from peripheral (e.g., brachial) pulse waveform recordings, it is possible not only to obtain aoBP levels (e.g., by applying a GTF or NPMA), but also to re-calibrate the peripheral (brachial) waveform to correct the under- and over-estimation of bSBP and bDBP, respectively, generated by measuring bBP with a cuff. Thus, by obtaining the aoBP and a corrected (re-calibrated) bBP, it is “theoretically” possible to quantify the center-peripheral SBPA or PPA more accurately (although this aspect requires further research). However, this is not possible if aoBP is simply quantified from CCA waveform recordings, as no peripheral waveform information will be available. Consequently, the best way to obtain aoBP (e.g., by CCA waveform analysis) is not necessarily the best approach (in practical terms) to quantify SBPA or PPA. Perhaps moving towards a “dual pulse recording” of peripheral and central pulse waveforms (as is done with various methods of calculating carotid-to-femoral or carotid-to-radial pulse wave velocity) is an accurate solution to simultaneously access reliable aoBP, bBP and SBPA (or PPA) measurements, although methodologically more complex.

### 2.3. The best way to calibrate the signals

**Figure 1:**
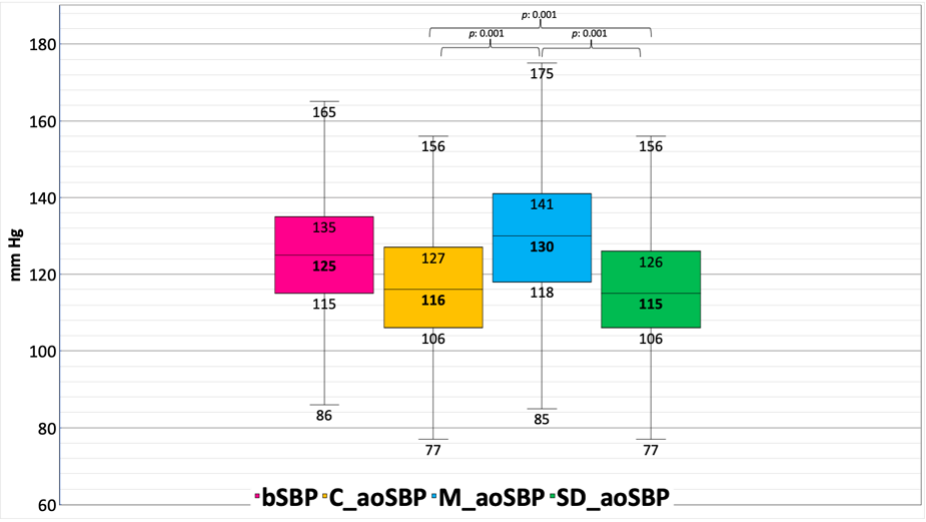
Differences obtained between different approaches and calibration methods for non-invasive measurement of aortic systolic blood pressure (aoSBP) in 1,654 subjects from a population-based study. **bSBP**: brachial systolic blood pressure. **C_aoSBP**: aortic systolic blood pressure determined by calibration to brachial diastolic, and mean blood pressure (bDBP, bMBP) calculated (CM) from bSBP and bDBP. **M_aoSBP**: aortic systolic blood pressure determined by calibration to bDBP, and (bMBP) measured by oscillometry (OscM). **SD_aoSBP**: aortic systolic blood pressure determined by calibration to bSBP and bDBP (termed systolic-diastolic or “SD”).

Regarding the third aspect, two different bBP-associated calibration methods have been mostly used: (i) calibration to bSBP and bDBP [systolic-diastolic (SD)], and (ii) calibration to bDBP and brachial MBP (bMBP) (5–8). bMBP levels to be used for calibration could correspond to bMBP measured by oscillometry (OscM) or calculated (CM) from bSBP and bDBP, using different scaling form factors (e.g., 33%, 40%, 41.2%) (5–7). Previous works analyzed whether aoSBP levels obtained with the same or different devices would be significantly modified by the calibration method considered (Figure 1) (6). Related to this, it should be noted that the calibration method that minimizes error when using a specific device or methodology may differ when using another approach. Our results and those of other authors suggested that the significant source of error when determining aoBP is the method of calibration used (5, 8, 9). For example, from BA recordings (oscillometry/plethysmography + GTF), the aoSBP levels were 123 ± 3 when using the calculated bMBP (FF = 33%) and 139 ± 4 mmHg using the oscillometric bMBP. As a further example, from CCA tonometry recordings, aoSBP levels were 120 ± 4 mmHg (bMBP quantified using an FF = 33%) and 135 ± 4 mmHg (oscillometricbMBP) (8). Additionally, we found that when calibrating the BP waveforms, using non-invasively measured bSBP and bDBP levels, an FF = 0.40 minimized the error between aoBP levels obtained non-invasively and invasively (8). Consequently, both the calibration method (e.g., bSBP/bDBP vs. bMBP/bDBP), as well as the way to obtain the bMBP (calculated vs. oscillometric), or even the best equation to get the bMBP (e.g., FF = 33% vs. FF = 40%) remains to be agreed. At least in theory, one aspect to be further evaluated is whether non-invasive aoBP measurements could be improved by using subject-specific FF levels (rather than applying a single FF to all subjects), quantified from central and/or peripheral waveform recordings. In summary, this demonstrates the importance of (i) being able to decide as soon as possible (consensus) on the FF that should be used to calibrate the aoBP records and (ii) (authors) communicating the method of calibration used. This is necessary to evaluate, analyze and adequately compare data from different studies or populations.

On the other hand, it is important that those responsible for developing technology (devices and software) leave open the possibility for the operator (researcher) to calibrate the records in different ways so that the best way of calibration can be investigated. In addition, it is important that oscillometric devices, which measure bMBP and then calculate bSBP and bDBP (and aoBP), allow the bMBP levels obtained with oscillometry to be visible (on the equipment's displays, and not only when downloading the information as text files).

The calibration method impacts whether potential differences in aoBP levels between different physiological or clinical conditions are minimized or maximized, which dramatically modifies the understanding of clinical entities (e.g., understanding whether aoSBP is affected or not in chronic pathologies [e.g., HIV infection] (10, 11), and whether it is affected to a lesser or greater extent than bBP). Consequently, the calibration method is “much more than the values obtained”, as it determines our understanding of the physiology and pathophysiology of aoSBP.

Considering the calibration-related differences between aoBP non-invasively and invasively obtained, non-invasive devices were categorized into two types based on function: Type I estimates “adequately” aoBP relative to measured bBP, and Type II estimates “adequately” intra-arterial aoBP (2). Schematically, this classification focuses on what we wish to know: (i) to correctly quantify the aoBP/bBP relationship (e.g., to assess SBPA and PPA), despite knowing that bBP levels have measurement errors (e.g., under- and over-estimation of bSBP and bDBP) (Type I), vs., (ii) to get closer to accurately knowing existing aoSBP levels, even though the relationship between aoBP (properly quantified) and bBP is distorted (e.g., aoSBP levels turn out to be higher than bBSP levels, which is generally not physiologically possible) (Type 2) (2). This schematic division has been an attempt to clarify that not all devices/approaches allow adequate quantification of aoSBP and SBPA. However, as was mentioned, future work will have to resolve how to properly quantify aoSBP and SBPA, with the same device, in the context of recognizing that the bBP measurements used to calibrate the signals present errors.

### 2.4. The existence of (poorly studied) proportional errors

A fourth aspect that should be discussed is that the devices show proportional error, which depends on the aoBP levels existing in the people evaluated (8). In general, the “overall” mean (or systematic) error shows that different devices, recording methods, or calibration methods tend to non-invasively quantify aoBP levels that are below the invasively recorded aoBP level (2, 8). Nevertheless, most approaches overestimated and underestimated aoSBP at low and high invasive aoSBP levels, respectively (8). Consequently, further work will be necessary not only to validate whether the devices present reduced global ´mean erroŕ levels concerning invasive recordings but also whether they allow adequate measurements to be achieved in a wide range of BP levels and, are useful precisely in patients in which records become essential when it comes to discriminating hemodynamic states (e.g., patients with high bBP levels, in whom it is desired to know their aoBP levels).

## 3. Biomedical impact of measurement controversies

The significant differences in the non-invasive determination of aoBP have clinical and biological/physiological implications. Concerning the former, up to now, many questions remain concerning the incremental predictive ability of aoBP over bBP, as well as the possible role of aoBP-guided therapy in everyday practice (12). These doubts are closely related to the “heterogeneity” in how aoBP is measured (e.g., aoBP may not outperform bBP in its predictive ability, given that there are calibration methods that “force a mathematical link between aoBP and bBP so that their independent predictive skills are not valued”). Additionally, the fact that aoBP values would not be obtained accurately could influence/distort the relationship between aoBP and cardiovascular risk levels; at the time, it could contribute to explaining differences in available data regarding the clinical value of aoBP in terms of risk stratification (8). For instance, it should be noted that frequently underestimated aoBP levels used to quantify left ventricular wall stress or arterial stiffness would lead to underestimation or overestimation of actual values (13). Then, understanding physiological or pathophysiological phenomena would be inaccurately evaluated (8).

An additional aspect that remains to be defined more precisely, and to reach a consensus, is the usefulness of aoBP non-invasive measurements in children and adolescents. Up to now, numerous pieces of evidence indicate that the measurement of aoBP in pediatric ages could be useful both to characterize physiological aspects (e.g., haemodyncamic changes during growth, sex-related haemodynamic differences) and/or to characterize clinical conditions (e.g., impact of arterial hypertension, obesity, etc., on central haemodynamics) (14–19). Everything indicates that also, in children and adolescents the information of the aoBP would be complementary to that of the bBP. However, the potential biomedical utility of measuring aoBP in pediatric ages is yet to be defined and agreed upon. Additionally, the relative impact that the four aspects previously analyzed have on the aoBP records in children and adolescents must be specifically evaluated since specific hemodynamic conditions are expected in them that can make the records more complex (e.g., lower levels of aoBP, higher heart rate), as well as technical difficulties specific to trying to record on smaller arteries (e.g., RA) and/or in places more difficult to access (e.g., ultrasound recordings in CCA).

## 4. Concluding summary

The recording method and site, the mathematical model used to quantify aoBP, and mainly the method applied to calibrate waveforms are essential when estimating aoBP (and SBPA or PPA) and should be considered when analyzing and/or comparing data from different works, populations, and/or obtained with different approaches. It is important to highlight the urgent need for methodological transparency and consensus for accurate and non-invasive assessment of aoBP, which would help increase its validity and value in clinical and physiological research.
